# Self-consistent Field Analysis of Segregative Aqueous Dextran–Polyethylene Glycol Solutions: (1) Bulk Phase Diagrams

**DOI:** 10.1021/acs.jpcb.5c01284

**Published:** 2025-06-19

**Authors:** F. A. M. Leermakers, L Ruiz-Martínez, S. D. Stoyanov, J. van der Gucht

**Affiliations:** † Physical Chemistry and Soft Matter, Wageningen University, Stippeneng 4, Wageningen 6708 WE, the Netherlands; ‡ Food, Chemical, and Biotechnology cluster, Singapore Institute of Technology, 10 DoverDrive, Singapore 138683, Singapore

## Abstract

Quasi-ternary dextran-water-polyethylene glycol (D-W-PEG) systems can undergo a segregative phase transition from a homogeneous to two-phase state with one phase enriched in dextran, the other in PEG, and both phases typically dominated by the spectator-like water component. The relation between the driving forces and the resulting phase behavior for such aqueous two-phase systems (ATPSs) is systematically studied with a Scheutjens–Fleer self-consistent field (SF-SCF) theory. We consider both the repulsive interactions between the polymeric species (the major driving force) and the solvent quality disparity (the minor driving force). In a narrow parameter range, when the major driving force is very weak, a closed-loop two-phase region with two critical points may be found. However, in more realistic parameter settings, an ATPS has an’open’ binodal with a single critical point. We report on the volume ratio, the composition of the phases, (structural) parameters of the interface, such as the width, and the interfacial tension, as a function of the (average) water fraction in the system. We show that the volume-management strategy influences the phase diagram when polymers are polydisperse. By fitting a set of experimental phase diagrams, SF-SCF model parameters for D-W-PEG systems are established, which will be used in future to underpin experiments for these systems.

## Graphical abstract

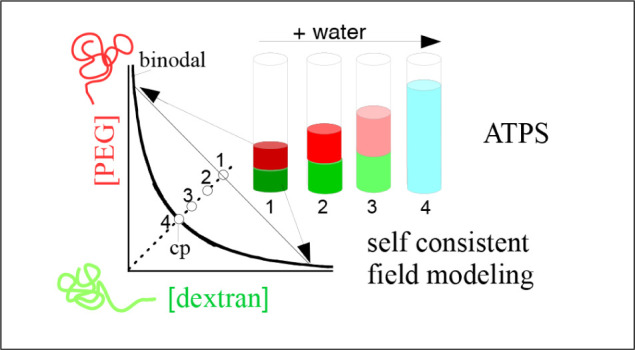

## Introduction

Ternary systems with two polymeric components in a common solvent, for example dextran and poly­(ethylene glycol) (PEG) in water, are well-known to undergo a segregative phase transition resulting in Aqueous Two-Phase Systems (ATPS) with one phase relatively rich in dextran, the other relatively rich in PEG, while both phases are particularly rich in the monomeric solvent, water.^1–8^ An ATPS is not only of interest from a fundamental perspective, because one can study phase behavior using a simple control parameter such as the amount of water in the system, but also from an applications perspective. They are useful for the separation of delicate proteins,^9–11^ or to give structure to foods.^12^ Moreover, these systems have potential for (bio)­material science.^13^

A polymer-based ATPS is usually a quasi-three-component system because polymers are rarely monodisperse. We will probe polydispersity effects, but first consider these systems as ideal ternary systems. Focusing on fluid phases, the Gibbs phase rule allows for three coexisting phases. However, when the poor solvent conditions are excluded, the system is in either a single homogeneous state or a two-phase state. There are various scenarios that lead to two phases. One can distinguish associative and segregative conditions.^14^

(i) *Associative phase behavior*. When the two polymers attract each other, they may form a coacervate phase which is rich in both polymers. The solvent is then expelled to the other phase, which is lean in typically both polymers. In a phase diagram, the coexisting concentrations of the two polymers are plotted. Then a closed-loop binodal with two critical points is expected wherein tie lines (lines in the phase diagram that connect coexisting phases) run from near the origin (systems high in water content and lean in both polymers) to the top-right corner, that is, a region with high polymer concentrations (low in water content). Typical examples are systems with oppositely charged polyelectrolytes.^15^ As opposite charges attract, the composition of the phase rich in both polymers is nearly charge compensated. Invariably, there is salt in such solutions and thus the solvent is a mixture of water and salts. Indeed, the salt (sometimes the pH) is the control parameter, meaning that with the salt concentration one can tune the degree of association. Coacervate droplets are relevant models for membraneless organelles in biological cells.^16^ In this paper, we will not consider this scenario in more detail.

(ii) *Segregative phase behavior* In this paper, we focus on another scenario. When the two polymers repel each other, the’major’ driving force, one also gets two phases, but now one type of polymer is segregated from the other; hence, each polymer partitions in its own separate phase. Then, typically, the solvent distributes over the two phases more or less evenly. The tie lines of the phase diagram run from top left to the bottom right. Now, the water content is the natural control parameter. When water is added, it dilutes the polymers. On average, this separates them from each other. Then the interactions between the two polymers will occur less often and this decreases the tendency to separate. We may refer to this phase diagram as being’open’ (in contrast to the closed-loop one discussed above), and the system has a single critical point. Even when the major driving force (repulsion between the two polymers) is very weak, there can still be segregative tendencies. Then it is possible (at least in theory) that this is orchestrated by a solvent quality disparity the’minor’ driving force. When there is a significant difference in solvent quality, meaning that one polymer likes water much better than the other one, again two phases form, but now one phase is more rich in water than the other one. The polymer that likes water best will predominantly be in the phase with more water and the other one will be in the phase that has a bit less water. The phase diagram has a closed-loop binodal (as in associative phase diagrams) with two critical points. One critical point is in common with the strong driving force cases, that is, the system loses its tendency to phase separate when the amount of solvent is increased above a critical value. On top of this, when the amount of solvent drops below some critical value, there is not enough water to facilitate two phases, giving the second critical point (by themselves the two polymers mix ideally in this scenario). Now, the system suffers a critical point with decreasing water content.

We may use the Flory–Huggins (dimensionless) mean-field mixing free energy density *f* = *F*/*k*_*B*_ *T J*, with *k*_*B*_ *T* the thermal energy, *J* the number of lattice sites (with characteristic length *b*), that is, the dimensionless volume, to learn about the trends discussed above. For a ternary system with one polymer-type composed of *N*_*D*_ segments of type D, another polymer with *N*_*P*_ segments of type P, and a monomeric solvent S, the (dimensionless) Flory–Huggins free energy density is given by^17^

1 $$\begin{array}{l} f=\frac{\varphi_{D}}{N_{D}}\ln\varphi_{D}+\frac{\varphi_{P}}{N_{P}}\ln\varphi_{P}+\varphi_{S}\ln\varphi_{S}+\\ \chi_{DP}\varphi_{D}\varphi_{P}+\chi_{DS}\varphi_{D}\varphi_{S}+\chi_{PS}\varphi_{P}\varphi_{S} \end{array}$$

where φ is the volume fraction (dimensionless segment concentration) and χ the Flory–Huggins interaction parameter which exists for unlike contacts: $\chi_{XY}=\frac{Z}{2k_{B}T}\left[2U_{XY}-U_{XX}-U_{YY}\right]$ , where *U* is the well-depth of a square-well potential, which has only a finite value for nearest neighbors. The logarithmic terms (cf. eq 1) correspond to (minus) the dimensionless mixing entropy. The final three terms represent the interactions in the system (again made dimensionless as the thermal energy *k*_*B*_ *T* is included in the χ parameters). One can reduce the number of volume fraction variables by one, because the volume fractions add up to unity: $\varphi_{A}+\varphi_{B}+\varphi_{S}=1$ (incompressible limit). The spinodal points may be found analytically, were the determinant of the free energy Hessian equals zero:

2 $$f_{DD}f_{PP}-f_{DP}^{2}=0$$

where *f*_*XY*_ ≡ ∂^2^ *f*/∂φ_*x*_∂φ_*Y*_. If the spinodal curve is a closed loop, the binodal curve is closed as well. Hence, there is a simple measure for distinguishing the open binodal from the closed one. The binodal points are usually found numerically, although in the vicinity of critical points there may be analytical approximations.

In the following, we will use the FH theory to characterize the phase diagrams, but we also employ the Scheutjens–Fleer self-consistent field (SF-SCF) approach.^18–20^ This method can be seen as an extension of the Flory–Huggins theory and gives the binodal compositions as well as the structure and thermodynamics of the corresponding interfaces between these phases. In the classical implementation of the SF-SCF scheme, the chains are assumed to be composed of (discrete) segments and space is discretized by a lattice such that segments fit exactly on lattice sites. The chain statistics follow the rules of the Freely-Jointed Chain (FJC) model, implemented on the lattice. Interactions are accounted for by the Bragg–Williams mean-field approximation^21^ parametrized by Flory–Huggins χ-parameters In such systems the incompressibility condition is applied, not over full space but at specified layers of lattice sites. Besides the binodal information, which we can already extract from the Flory–Huggins approach, we also find information on, e.g., the density profiles across the interface, the width of the interface and thermodynamic quantities such as the interfacial tension. In the Supporting Information, we give a more detailed description of the SF-SCF method.

Typically, we will assume that the interface is planar so that results will apply to systems that feature macroscopic phase separation. As in a particular computation the system size (system volume) must be specified and necessarily is finite, it is clear that calculations sufficiently close to the critical point will fail as soon as the width of the interface is comparable to the system size. So, we usually do not have an accurate estimate of the critical point using this method. In SF-SCF calculations it is possible to treat components in a grand-canonical way. More specifically, this option is used for the solvent (water) component. We fix the concentration of water in the bulk (typically the PEG-rich phase is used for this) and compute the corresponding concentration in the dextran-rich phase. Then, the amount of solvent (water) in the system is not fixed and may change (as if it is in equilibrium with a reservoir). This means that the results for pure ternary systems do not depend on the volumes of the phases (unless the interface is close to the system boundaries). The same computational strategy could not be implemented for polymers that are polydisperse. As will be shown, one consequence of this is that various system properties, such as, the phase diagrams, are affected by the phase volume strategy that is implemented in the case of polydispersity.

The results section will be split up into several subsections. We will start with closed-loop phase diagrams, for close-to-symmetric ternary systems that have very weak polymer–polymer interactions and sufficiently strong solvent quality differences. This is followed by subsections devoted to the open-binodal cases (sufficiently strong polymeric interactions and/or relatively minor solvent quality differences), and discuss how the solvent quality disparity and polydispersity affect the phase diagrams. In the final section, we present our experimental phase diagram and report on our attempts to fit it. In the discussion we will elaborate on remaining theoretical and experimental issues.

## Results

Even though the phase diagrams may be extracted from the numerical analysis of the Flory–Huggins free energy density, we typically present those obtained from SF-SCF computations because in this approach we also obtain structural and thermodynamical data of the interfaces in the system. In some cases, we will also report (analytical) spinodal curves. These are found from FH theory. In the phase diagrams, we put the PEG concentration on the *y*-axis and the dextran concentration on the *x*-axis. The PEG component is chosen to be monodisperse. The PEG component typically has the lower molecular weight, and/or has the better solvent quality of the two. The dextran component may have a molecular weight distribution. However, we first treat the systems as purely ternary (and monodisperse).

### Closed-Loop Phase Diagrams

Let us first consider a rather academic parameter setting, namely, that the two polymers mix ideally χ_*DP*_ ≈ 0, while both polymers have significantly different interactions with the solvent (water) 0 < χ_PS_ < χ_*DS*_ ≤ 0.5. In this segregative situation, a closed-loop phase diagram is found. As PEG and dextran are used here as’generic’ names, we can choose χ-values freely. Only when the experimental phase diagram is fitted below, we will restrict the parameters to relevant ones for the PEG-water-dextran system. The solvent was treated grand canonically,^21^ which implements that solvent can diffuse into or out of the system as it is in equilibrium with a reservoir (with a composition similar to the PEG-rich phase). The position of the interface is controlled by specifying the amount of dextran in the system (canonical computations^21^). The exact location of the interface is irrelevant for the phase diagram unless it is too close to the system boundary. We carefully avoided these pathological cases.

A typical example for the volume fraction profiles across the interface for segregative phase behavior is presented in Figure 1a; panel b) will be discussed below. In panel (a) the polymers do not repel each other; they mix ideally. The solvent quality for PEG is slightly better than that for dextran. This difference is enough to drive the segregation. In this case, the position of the interface is set at *z* = 0 and dextran is predominantly found at negative *z*-coordinates (phase α), while most of PEG is mostly at positive *z*-values (phase β). The solvent density is, as anticipated, noticeably higher at high *z*-values, than at low ones. In this graph, we use the opportunity to illustrate how the width of the interface is estimated. This is done by finding the highest slope of the density profile *s*_max_ ≡ (∂φ_*D*_/∂z)_max_, which occurs typically halfway the interface, and then *W* = Δφ_D_/*s*_max_, where Δφ_*D*_ = φ_*D*_(−∞) – φ_*D*_(∞). The density difference across the interface for the solvent is found similarly.

**Figure 1:**
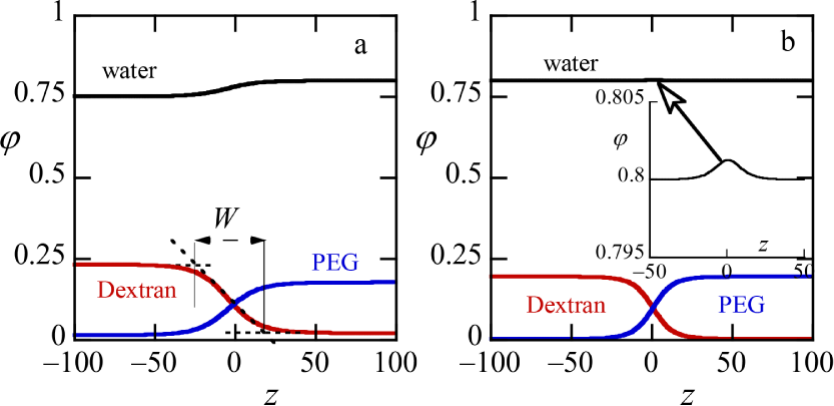
Volume fraction profiles for the dextran (D), PEG, (P) and water components across the interface. *N*_*P*_ = 1000 and *N*_*D*_ = 1000. (a) Segregation driven by a solvent quality difference: χ_*DP*_ = 0, χ_*DS*_ = 0.5, χ_*PS*_ = 0.4. (b) Segregation driven by polymer–polymer repulsion: χ_*DP*_ = 0.02, χ_*DS*_ = χ_*PS*_ = 0. The inset in panel (b) shows the (small) density maximum of the solvent at the interface. In panel (a), the computation of the width *W* of the interface is illustrated. In short, the slope (at the midpoint of the interface) is extrapolated to the bulk values to find *W*.

In Figure 2 we present typical results for systems with a closed-loop segregative phase diagram; the parameters are similar as in Figure 1a: both polymers have the same degree of polymerization *N*_*P*_ = *N*_*D*_ = 1000. The interactions between the polymers is athermal χ_*PD*_ = 0 and there is a 0.1 difference between the two solvent qualities χ_*DS*_ = 0.5 and χ_*PS*_ = 0.4. In panel (a) both the binodal (solid line) and spinodal (dotted line) are presented. These curves meet at both critical points. The lower critical point in terms of volume fraction of solvent is $\varphi_{S}^{cr}\approx 0.430575$ and the upper one at $\varphi_{S}^{cr}\approx 0.9301$ . The tie lines have a slope of −1 (not shown). The phase diagrams of this type have a triangular shape with three corners: close to the origin, top left and bottom right. The critical point at low water content is bounded by $\varphi_{P}^{cr}+\varphi_{D}^{cr}=1$ . The top left corner can approach­(φ_*D*_, φ_*P*_) = (1, 0) in the limit of large solvent quality disparity and very high molecular weights. In the same limits, the bottom right corner approaches (1,0). The lower left corner will be studied below more specifically for the’open’-binodal cases. The phase diagram is slightly asymmetric and this is obviously caused by the solvent quality difference. As dextran is in the poorer solvent, it is seen that the dextran-rich phase is lower in water content than the PEG-rich phase.

**Figure 2:**
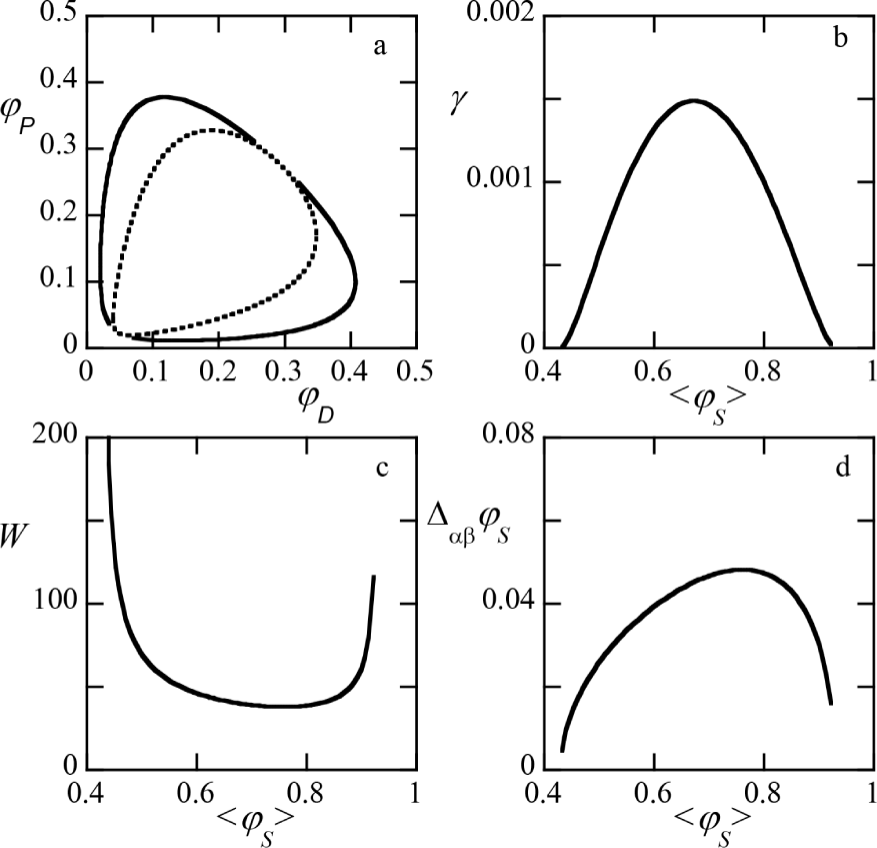
(a) Phase diagram binodal (solid line) and spinodal (dotted line) for ATPS. Here on the *y*-axis is the volume fraction of dextran φ_*D*_ and on *x*-axis we plotted the corresponding value of PEG φ_*P*_. Parameters: *N*_*D*_ = *N*_*P*_ = 1000 *N*_*S*_ = 1 χ_*PD*_ = 0, χ_*PS*_ = 0.4 and χ_*DS*_ = 0.5. (b) Corresponding interfacial tension γ in lattice units (*k*_*B*_ *T*/*b* ^2^, with *b* is segment length), (c) interfacial width *W* in unit *b*, d) difference in volume fraction of solvent $\Delta_{\alpha \beta }\varphi_{S}≡\left|\varphi_{S}^{\alpha }-\varphi_{S}^{\beta }\right|$ between the two phases (α (dextran-rich) and β (PEG-rich)), (b–d) all plotted as a function of the average volume fraction of solvent $\left\langle \varphi_{S}\right\rangle ≡\left(\varphi_{S}^{\alpha }+\varphi_{S}^{\beta }\right)/2$ . The lower critical volume fraction of solvent $\varphi_{S}^{cr}\approx 0.430575$ and the higher critical volume fraction of solvent $\varphi_{S}^{cr}\approx 0.9301$ .

The remaining panels in Figure 2 give the interfacial tension (see the Supporting Information for how this quantity is evaluated), the width of the interface (cf. Figure 1a) and the solvent volume fraction difference as a function of the average volume fraction of solvent in the system (that is, the average over the two phases). We see that the interfacial tension vanishes at both critical points and there is a maximum in between. The interfacial width diverges at both critical points and goes through a minimum in between. Finally, the solvent density difference in both phases passes through a maximum and vanishes at the critical points. It is immediately clear from these results that such systems are typically in a weak segregation state. The interfacial tension (expressed in dimensionless units) is 10^–3^ or less. With a segment length *b* ≈ 0.5× 10^–9^ m, the conversion factor to mN/m is about 16, and thus the interfacial tension is ≈ 10^–2^ mN/m or lower. In line with this, the interfacial width exceeds *W* = 50b and is significantly larger than the coil size *R* ∝ *bN* ^1/2^. Even in this weak segregation limit the differences in amount of solvent can be as large as 5%.

We can define $\Delta \varphi_{S}≡\left|\varphi_{S}^{cr}-\left\langle \varphi_{S}\right\rangle \right|$ as the difference of the average volume fraction of solvent in relation to the (closest) critical volume fraction of solvent. Approaching either the lower or the higher critical point, we find power-law dependencies with the expected mean-field exponents:^22^ $\gamma \propto \Delta \varphi_{S}^{3/2}$ , $W\propto \Delta \varphi_{S}^{-1/2}$ , and $\Delta_{\alpha \beta }\varphi_{S}\propto \Delta \varphi_{S}^{1/2}$ . In fact, the critical points that were presented above for this system were selected as those values that gave the best fit with the presented (expected) mean-field power-law coefficients. We will return below to these power-law scaling dependencies and discuss these in more detail.

A selection of closed-loop phase diagrams is presented in Figure 3, each parameter setting has its own color. Again, binodals are plotted in solid lines and spinodals are dotted. The black curves (panels a, c, d) are the same as in Figure 2a. The dark blue curves in Figure 3a,b are also copies.

**Figure 3:**
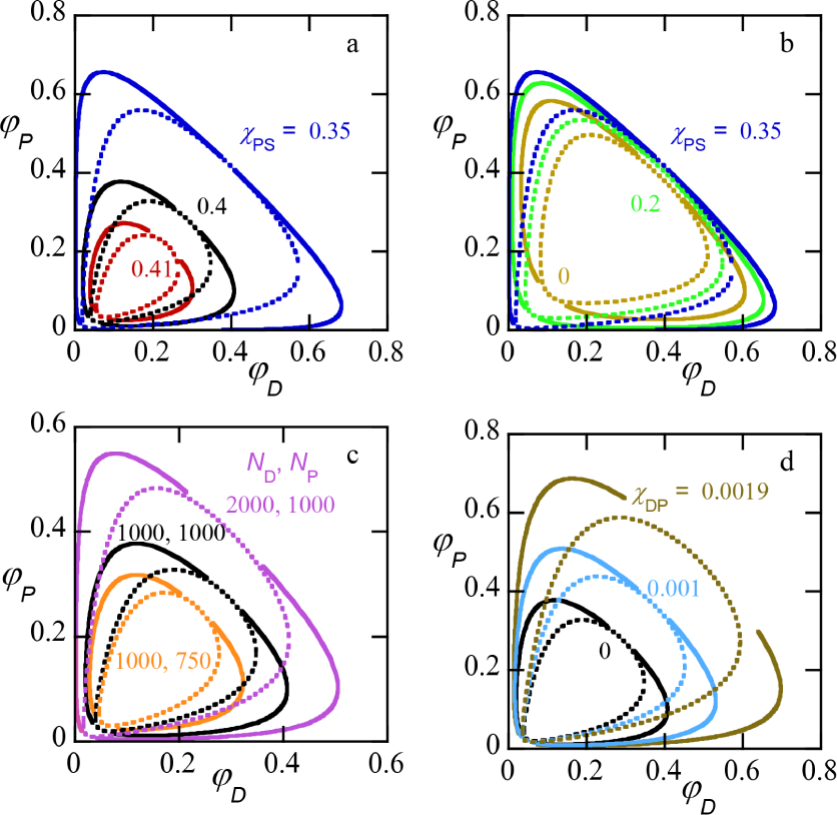
Collection of closed-loop phase diagrams for ATPS systems: binodal (solid lines) and spinodal (dotted lines). (a) Effect of χ_PS_ as indicated. (b) For Fixed difference χ_*DS*_ – χ_*PS*_ = 0.14 while the solvent qualities are varied as indicated. (c) The effect of molecular weights of the two polymers as indicated. (d) The effect of χ_*DP*_ as indicated. Default parameter as in Figure 2, i.e., *N*_*D*_ = 1000, *N*_*P*_ = 1000, χ_*DP*_ = 0, χ_*DS*_ = 0.5 and χ_*PS*_ = 0.4.

In Figure 3a,b the influence of the solvent qualities is presented. Whereas in panel (a) the value of χ_*DS*_ = 0.5, in (b) the difference χ_*DS*_-χ_*PS*_ = 0.15 is kept constant. Both the difference in solvent quality and the absolute values are relevant. From panel (a), it is easily seen that with decreasing χ_*PS*_ the phase diagram widens and with increasing value (that is, when the difference in solvent quality reduces) the phase diagram shrinks. Focusing on the critical points, as a function of χ_*PS*_, we may find two lines of critical points that meet when χ*_PS_* ≈ 0.4187, and the phase diagram has shrunken to a point. From panel b), we understand that the poorer the solvent quality is, the wider is the phase diagram. In the following, we will see that the absolute value of the solvent qualities also matters for the open-binodal cases. In panel (c) we present trends with respect to the chain length of the two polymers for given solvent quality parameters (χ_*DS*_ = 0.5 and χ_*PS*_ = 0.4). As expected, the larger the chains, the wider the phase diagram. A length difference between the polymers will influence the asymmetry of the phase diagram as well. Long chains have less mixing entropy than short ones, and therefore, the solvent prefers the phase with the shorter chains slightly. In this case, the length of the dextran chains was taken longer than that of the PEG chains, and thus the length difference of the chains added to the asymmetry of the phase diagrams. Only when the length of the PEG would have been larger than that of dextran could we have found phase diagrams that are close to symmetric as chain length differences and solvent quality differences could have compensated for each other. For given solvent strength parameters one can once again find lines of critical points as a function of, e.g., the chain length of PEG (fixed length of dextran). For short enough PEG chains the phase diagram may vanish again in a multicritical point.

Experimentally more interesting, in Figure 3 we present phase diagrams for different values of χ_*DP*_, while other parameters such as the solvent quality and chain lengths were fixed. It is quite natural to expect that χ_*DP*_ is finite and positive. With increasing values of the repulsion between the two polymers, the phase diagram widens. The critical point on the polymer-rich side (lean in solvent) quickly approaches the limiting value of (0.5, 0.5). When there is no solvent, the critical point is given by χ_*PD*_ *N* = 2. For *N* = 1000, the value of χ_*PD*_ = 0.002 is the last closed-loop binodal value. This means that when χ_*PD*_ > 0.002 phase diagrams will be of the open-binodal type. In the remainder of this paper we will discuss open-binodal systems, and we often will drop the word’open’ as it will be clear what type of phase diagram is considered.

### Open Binodal: Athermal Solvents

Often when ATPS phase diagrams are considered, the solvent quality is ignored: the parameters that define the solvent quality are simply set to zero, that is, χ_*DS*_ = χ_*PS*_ = 0. In this subsection, we choose this athermal solvent setting as well, even though it is clear that in real systems the solvent quality should be included.

The (dimensionless) Flory–Huggins free energy density now reduces to

3 $$f=\frac{\varphi_{D}}{N_{D}}\ln\varphi_{D}+\frac{\varphi_{P}}{N_{P}}\ln\varphi_{P}+\varphi_{S}\ln\varphi_{S}+\chi \varphi_{P}\varphi_{D}$$

Here, χ ≡ χ_*PD*_, which is not confusing because both χ_*PS*_ = χ_*DS*_ = 0 in this case.

The critical conditions are found by solving for the root of the second and third derivatives:

4 $$\varphi_{D}^{cr}=\frac{\sqrt{N_{P}}}{\sqrt{N_{P}}+\sqrt{N_{D}}}\left(1-\varphi_{S}^{cr}\right)$$

5 $$\varphi_{P}^{cr}=\frac{\sqrt{N_{D}}}{\sqrt{N_{P}}+\sqrt{N_{D}}}\left(1-\varphi_{S}^{cr}\right)$$

6 $$\varphi_{S}^{cr}=1-\varphi_{P}^{cr}-\varphi_{D}^{cr}$$

7 $$\chi^{cr}=\frac{1}{2}{\left(\frac{1}{\sqrt{N_{P}}}+\frac{1}{\sqrt{N_{D}}}\right)}^{2}/\left(1-\varphi_{S}^{cr}\right)$$

with $\varphi_{S}^{cr}=1-\varphi_{D}^{cr}-\varphi_{P}^{cr}$ . When both chains are equally long, i.e., *N* ≡ *N*_*D*_ = *N*_*P*_, an analytical approximation for the binodal (valid near the critical point) after Taylor expansion of the logarithmic terms, may be given as a function of the volume fraction of the solvent (at the binodal):

8 $$\varphi_{D}^{bin}=\frac{1-\varphi_{s}^{bin}}{2}\left(1\pm \sqrt{\frac{3}{2}\left(\chi N\left(1-\varphi_{s}^{bin}\right)-2\right)}\right)$$

where the plus and minus branches bracket the critical point and the volume fraction of PEG may be found from $\varphi_{P}^{bin}=1-\varphi_{S}^{bin}-\varphi_{D}^{bin}$ .

In Figure 1b a typical density profile is shown across an interface, for the case that segregation is driven by the major driving force. It is assumed that the polymers mix ideally with the solvent (athermal solvents). Again, the interface is placed at *z* = 0 and the dextran-rich phase is at lower, while the PEG-rich phase is at higher coordinates. The profiles have a tanh-like shape, known from the van der Waals theory.^22,23^ Interestingly, the solvent profile shows a small maximum as illustrated in the inset of Figure 1b. This adsorption peak is caused by the desire of the system to minimize the unfavorable PEG-dextran contacts.

Some typical phase diagrams are presented in Figure 4a. The binodal (solid lines) and spinodal (dotted lines) are completely symmetric when *N*_*P*_ = *N*_*D*_. Furthermore, the reduced binodal curves, when $\varphi_{P}/\varphi_{P}^{cr}$ is plotted versus $\varphi_{D}/\varphi_{D}^{cr}$ , fully overlap. This does not only occur when χ is varied, but also when *N* is changed. This is not too surprising, because upon inspection of eq 3 it is seen that the combination *χ N* is the only relevant parameter. When there is a difference in chain lengths, the ratio of critical densities obeys the relation $\varphi_{D}^{cr}/\varphi_{P}^{cr}=\sqrt{N_{P}/N_{D}}$ . This means that when *N*_*D*_ > *N*_*P*_, which is true in our experimental system (see below), the critical point is expected to be on a dilution line with slope larger than unity.

**Figure 4:**
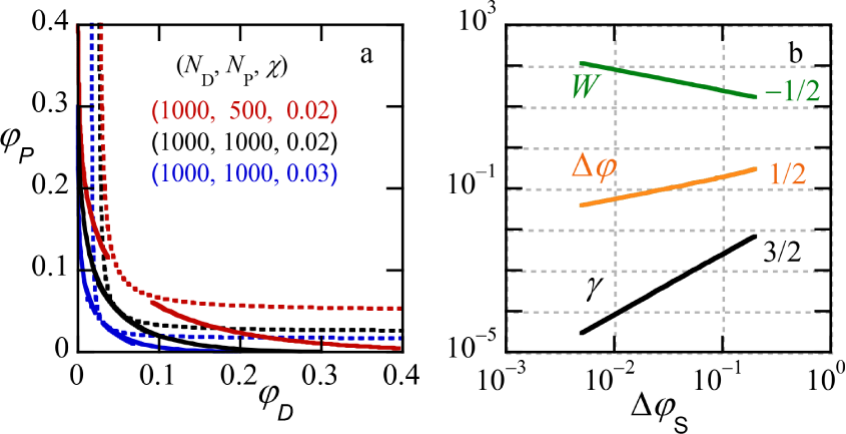
(a) Phase diagrams for ATPS with two (repulsive) polymers in a athermal solvent. Solid lines: binodal. No SF-SCF-results are generated for interfaces that are wider than *W*≈ 100b, which is why the binodal lines near the critical point are missing. However, for *N*_*P*_ = *N*_*D*_ the analytical solution 8 interpolates this part; dotted lines: spinodals. Other parameters are indicated in the graph. (b) The interfacial tension γ, the width *W* of the interface and the density difference Δφ ≡ |φ_*D*_ – φ_*P*_| (in either one of the bulk phases) in lattice units, as a function of $\Delta \varphi_{S}≡\varphi_{S}^{cr}-\varphi_{S}$ (where φ_*S*_ is a value in the bulk) in double logarithmic scale. The slopes are indicated near the curves. Parameters *N*_*P*_ = *N*_*D*_ = 1000, χ = 0.02.

As mentioned earlier numerical SF-SCF data near the critical point are expected to fail because the width of the interface diverges. Typically we therefore show results up to interfacial widths of order *W* ≈ 100 lattice units. For the symmetric case, there is the analytical solution that works well in the range where the SF-SCF results are missing. That is why in Figure 4a the binodals continue and include the critical point. The spinodal and binodal lines meet at the critical point. Far from the critical point, the spinodals run (almost) parallel to the axis. Hence, there is a reasonable range of conditions in which we expect a nucleation and growth mechanism for phase separation.

In Figure 4b, results are shown for the interfacial tension (in units *k*_*B*_ *T*/*b* ^2^), the interfacial width *W* (in units b) and the volume fraction difference between the PEG and dextran component in any of the two phases, as a function of $\Delta \varphi_{S}≡\varphi_{S}^{cr}-\left\langle \varphi_{S}\right\rangle$ . Here we have chosen a pure symmetric case, *N*_*P*_ = *N*_*D*_ = 1000 and χ = 0.02 and the solvent is an ideal’spectator’ component. The density of solvent is the same in both phases and ⟨φ_*S*_⟩ =φ_*S*_, where it is understood that the volume fraction of S is taken somewhere in the bulk (far from the interface). Straight lines in Figure 4b imply power-law scaling and slopes are expected from van der Waals theory.^22^ It shows that φ_*S*_ is a useful control parameter in this system (comparable to the temperature *T* in classical phase separation studies).

It is important to estimate the critical point in a phase diagram. Using the critical point, we can estimate various quantities in the system, exemplified by the data of Figure 4b. One of the ways to find a critical point is to measure the interfacial tension and then search for the critical value of the control parameter such that a straight power-law curve is obtained. Another way to find the location of the critical point is to study the volume ratio of the phases along a dilution line. The dilution lineφ_*P*_ =*a*φ_*D*_ will go through the critical point where the slope is given by $a=\sqrt{N_{D}/N_{P}}$ (athermal solvents). When the phase diagram is known, it is possible to compute the volume ratio of the phases using the well-known lever rule.^24^

The fraction of volume taken up by the dextran-rich phase is given by *F*_*D*_ and is plotted as a function of the amount of solvent in the system in Figure 5. In panel (a) an exactly symmetric phase diagram is used. In this case, the tie lines are perfectly parallel and have a slope of −1. Hence, the dilution line that goes through the critical point has a slope of 1 and then *F*_*D*_ is perfectly flat until the critical volume fraction of solvent is reached (in this case $\varphi_{S}^{cr}=0.9$ ). Any other dilution line will bend upward and go to unity, or downward and go to 0 reaching this value exactly when the dilution line crosses the binodal. Unfortunately, such ideal dependencies for *F*_*D*_ are not always found.

**Figure 5:**
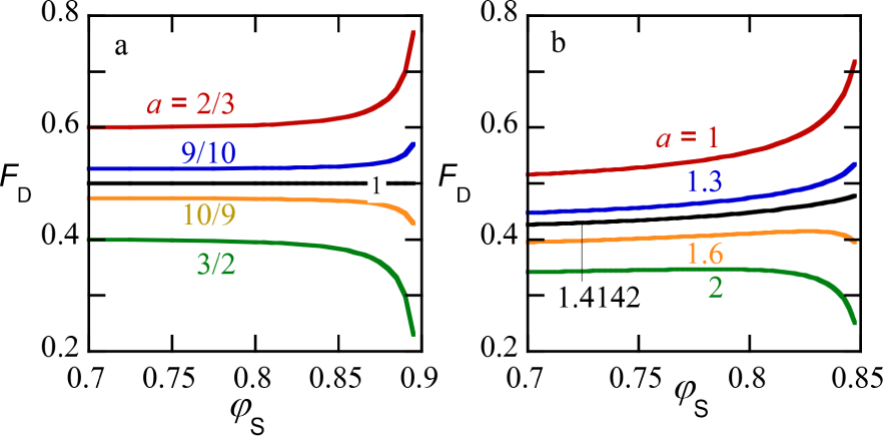
Fraction of the volume *F* taken by the dextran-rich phase, as a function of the (average) concentration of solvent $\varphi_{S}=F\varphi_{S}^{\left(D\right)}+\left(1-F\right)\varphi_{S}^{\left(P\right)}$ along a dilution line φ_*P*_ = *a*φ_*D*_. The slope values *a* are indicated near the graphs. The black line is the dilution line through the critical point. (a) Ideally symmetric system *N*_*P*_ = *N*_*D*_ = 1000, χ = 0.02 and (b) asymmetric phase diagram *N*_*P*_ = 500 *N*_*D*_ = 1000 and χ = 0.02.

When there is a difference in chain lengths between the polymers, important deviations are found. As can be seen in Figure 5, no dilution line is horizontal; they all start with a positive slope. The dilution line that goes through the critical point has a slope of $a=\sqrt{2}$ and reaches the limiting value of *F*_*D*_ = 0.5 when the critical point is reached; the slope of the dilution line $a>\sqrt{(}2)$ all *F*_*D*_(φ_*S*_)-curves will bend upward. Similarly, for dilution lines with slope $a>\sqrt{(}2)$ the *F*_*D*_(φ_*S*_)-curves bend downward.

There always exists a *F*_*D*_(φ_*S*_)-curve that has 0.5 as its limiting value. This curve defines the dilution line that goes through the critical point. This nice feature may be traced to the tangent of the binodal at the critical point. This tangent follows the binodal for a small part around the binodal and the corresponding tie line has equal lengths on both sides of the binodal. Hence, according to the lever rule, *F*_*D*_ → 0.5 a the critical point.

### Open Binodal: Effect of Solvent Qualities

Water is a complex fluid. Each water molecule is involved in 4 hydrogen bonds forming an interconnected, yet fluid, network. Polymers invariably disrupt this network and this is why they are reluctant to dissolve in it. This means that the ideal mixing conditions used in the previous paragraph are rarely met. Most often, water is a marginal or poor solvent for polymers. It is expected that the solvent quality for PEG is not far from theta conditions.^25^ Obviously, phase diagrams depend on the solvent quality.^26,27^ There exists little reliable information about the expected χ_*DS*_, that is, the solvent quality of water for dextran. This is in part due to the fact that dextran of biological origin is branched and more compact than linear macromolecules; hence, it is not straightforward to use light scattering to relate size to molecular weight. The PEG chains are linear and there are estimates in the literature that point to a value of approximately χ_*PS*_ ∼ 0.45.^25^ In practice it is known that PEG dissolves more easily in water than dextran. In part, this may be due to the difference in molecular weight, but we believe that this more specifically indicates χ*_DS_> χ*_*PS*_. In this section we will fix χ_*DS*_ = 0.5 and vary the solvent quality for the PEG chains to illustrate the role of the solvent quality (difference) on the phase diagrams.

In the closed-loop phase diagrams discussed above, it was evident that a difference in solvent strength for the two polymers amplify the tendency to segregate. This is illustrated in Figure 6 where a few phase diagrams for ATPS are shown with equal chain lengths *N*_*D*_ = *N*_*P*_ = 1000, a repulsion between the polymer segments χ_DP_ = 0.02, and for water being a θ-solvent for dextran. The critical point shifts to lower values of polymer concentrations for lower χ_*PS*_, that is, when the solvent quality of the PEG is improved. In this case, not only the repulsion between the polymers (parametrized by χ_*DP*_ = 0.02) is a driving force, but the difference in solvent quality χ_*DS*_ – χ_*PS*_ also contributes. Note that the black binodal, for which χ_*DS*_ = χ_*PS*_ = 0.5 is the same as the binodal in Figure 4 for χ_*DS*_ = χ_*PS*_ = 0.0. In both cases, the solvent is a spectator and has equal concentration in both phases. The absolute value of the solvent quality is in this symmetric setting immaterial.

**Figure 6:**
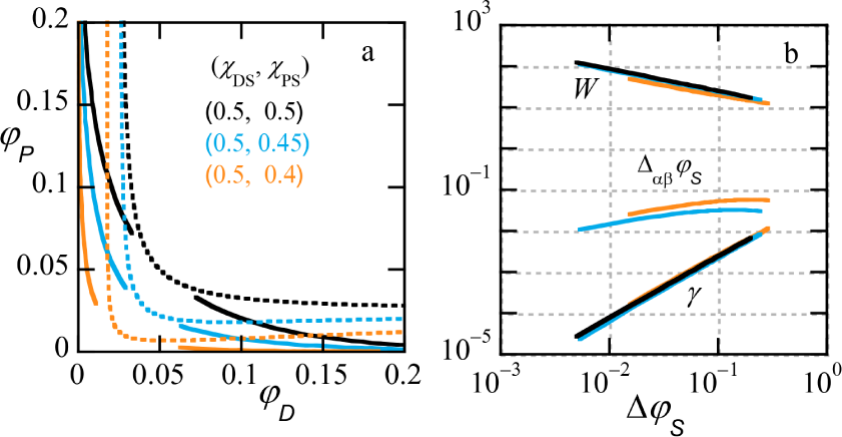
(a) Phase diagrams for ATPS (volume fraction of PEG φ_*P*_ vs volume fraction of dextran φ_*D*_), effect of solvent qualities: binodals (solid lines), spinodals (dotted lines). χ_*PS*_ is varied as indicated. (b) Corresponding scaling behavior for the interfacial tension of the interface (lattice units), the width of the interface and the density difference of the solvent between the two phases, as a function of Δφ_*P*_ on double logarithmic scale. Color code is the same as in panel (a). Other parameters: *N*_*D*_ = *N*_*P*_ = 1000, χ_*PD*_ = 0.02.

In line with the lowering of the critical point, the binodal lines run relatively close to the *x* and *y*-axis; indicating strong segregation of the two polymers, even when the volume fraction of water is of order 0.85. Importantly, in these phase diagrams, the tie lines are not at a slope of −1 nor do the tie lines run parallel. We will return to this effect below. The spinodal lines (dotted lines) basically resembles the athermal solvent case.

In Figure 6b we collect the interfacial width, the interfacial tension, and the difference in solvent volume fraction between the two phases (dextran-rich phase is referred by α and the PEG-rich one by β). These quantities are plotted as a function of $\Delta \varphi_{S}=\varphi_{S}^{cr}-\langle \varphi_{S}\rangle_{\alpha ,\beta }$ in double logarithmic scale. In these coordinates, there is little effect of the solvent quality difference, but it must be noted that the critical points are different and for specified volume fraction of solvent the quantities do differ noticeable with respect to the solvent quality. The slopes of the dependencies again closely obey the (mean field) expectations.

In Figure 7a we present the slope of the tie line *b* as a function of the (phase-averaged) solvent concentration in the system. The vertical dotted line is placed at the critical value of the solvent concentration. Not surprisingly, when χ_*PS*_ = 0.5, that is, when it is equal to the value of χ_*DS*_, the slopes are *b* = −1, and the tie lines run parallel. However, when there is a solvent strength disparity, the tie lines have a slope that is less negative and has the tendency to be even less negative when the critical point is reached. This means that the tie lines no longer run parallel. The extrapolated value of the slope at the critical point is indicated by the horizontal dotted lines. The smaller the solvent quality disparity, the closer this’final’ slope is to −1. More specifically, for χ_*PS*_ = −0.45, *b* ≈ −0.67, and for χ_*PS*_ = −0.45 we find *b* ≈ −0.45.

**Figure 7:**
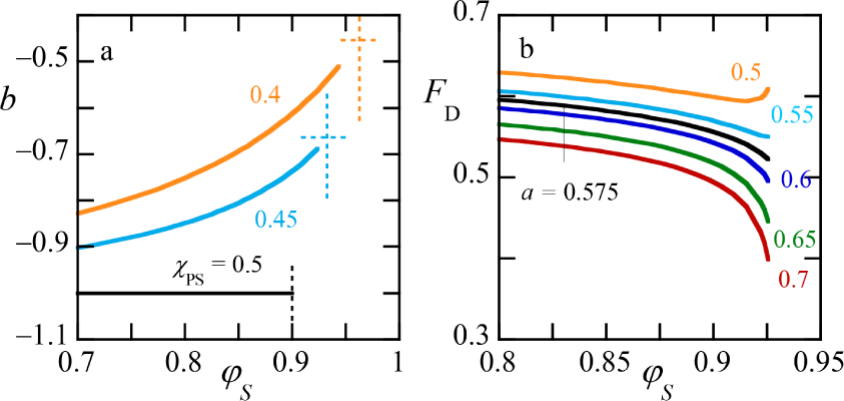
(a) The slope of the tie line $b≡\frac{\varphi_{P}^{\alpha }-\varphi_{P}\beta }{\varphi_{D}^{\alpha }-\varphi_{D}\beta }$ as a function of the volume fraction of solvent. The value of χ_*PS*_ is indicated. Vertical dotted line is placed at the critical value for the solvent volume fraction. The horizontal dotted line indicates the estimate for *b* at the critical point. The fraction of volume taken up by the dextran-rich phase as a function of the volume fraction of solvent χ_*PS*_ = 0.45. The value *a* of the tangent of the dilution line is indicated. The black curve is going (close) to the critical point as it approximately has the limit of *F*_*D*_ = 0.5. Other parameters: *N*_D_ = *N*_*P*_ = 1000, χ_*PD*_ = 0.02, χ_*DS*_ = 0.5.

Measurements of the phase volumes for given dilution lines are experimentally used to find the critical point. In Figure 7b we show the fraction of the volume occupied by the dextran rich phase *F*_*D*_ as a function of the volume fraction of solvent φ_*S*_ for different values of the slope of the dilution lines *a*. The black line in Figure 7b is the estimate for the’ideal’ dilution line that goes through the critical point (it has the limiting value of 0.5).

### Open Binodal: Effect of Polydispersity

Invariably synthetic and most biological macromolecules are polydisperse. Studying the effect of polydispersity on the ATPS phase diagrams hardly needs further motivation. In the literature there are several reports that consider polydispersity issues.^3,8,28–34^ When the focus is on the clouding curve and the composition of the corresponding shadow phase (the minority phase that emerges when the system passes the cloud point), it is known that a continuous distribution of chain lengths may be replaced by a limited number of chain lengths, provided that the relevant moments of the size distribution are similar.^29–31^ In polydisperse systems the composition of coexisting phases will change when the volume ratio of the phases is altered. That is why in the moment analysis the focus is on the situation that the volume of the minority phase is negligible compared to the majority phase. Then the composition of the majority phase is equal to the parent distribution and the composition of the shadow phase is not affected by compositional drift effects.

Cloud point titrations are a popular way to experimentally find estimates for the phase boundaries. Mimicking this with computations is not straightforward. It calls for grand-canonical calculations wherein the composition of the majority phase is fixed to the parent distribution, but this runs into the problem that the overall concentration is a priori unknown. Alternatively, one can perform canonical calculations, and fix the composition of the overall system to the parent distribution. The overall amount can be adjusted such that the volume of the majority phase is much larger than that of the minority phase. This ensures that the composition of the majority phase cannot drift away much from the parent distribution. However, such calculations become problematic when the interfaces are wide, that is, when the system is not far from its critical point. In this limit the minority phase cannot be taken small or else the interface is too close to the system boundaries. The volume of the majority phase must then be proportionally larger with concomitant computational challenges.

Below we will not attempt to probe the cloud- or shadow curve exactly. Instead, we set out to show how the volume ratio of the coexisting phases influences the binodal. We implemented canonical calculations and focused on manageable volume ratios. We do this for a number of arbitrarily chosen chain-length distributions. These distributions are restricted to a small number of chain lengths. This choice is in line with the moment approach which advocates that it is possible to find realistic results for a finite number of chain lengths provided that the moments remain similar to the continuous distributions.

To study the signature of polydispersity on the phase diagram, we choose to introduce polydispersity effects only for one of the two polymeric components. As the dextran component is of biological origin, it is more polydisperse than PEG. This is why we will keep the PEG monodisperse and set *N*_*P*_ = 550 and only consider the dextran component to be composed of a set of chains with different molecular weights. More specifically, we have introduced (arbitrarily) ten different lengths *N*_*D*_ = 100, 200, ···, 1000 and number these *j* = 1, 2, ···, 10. The relative amount (segment weight) θ_*j*_ differs in the following three cases. (i) The *flat* distribution: In this case θ_*j*_ = θ/10, where θ is the total amount of (segments) of dextran chains. The number-averaged and weight-averaged chain lengths are (*N*_*n*_, *N*_*w*_)=(341.4, 550). Hence, the polydispersity is *I* ≡ *N*_*w*_/*N*_*n*_ = 550/341.4 ≈ 1.61. (ii) The *linear* distribution: $\theta_{j}=\frac{11-j}{550}\cdot \theta$ and ((*N*_*n*_, *N*_*w*_) = (247.5, 400), *I* ≈ 1.62). (iii) The *bimodal* distribution with the lower molecular weight’peak’ twice higher than the high molecular weight one: θ_*j*_ = (2/33, 4/33, 10/33, 4/33, 2/33, 1/33, 2/33, 5/33, 2/33, 1/33)·θ and (*N*_*n*_, *N*_*w*_) ≈ (326, 467), *I* ≈ 1.43).

To highlight the polydispersity effect, we will avoid the solvent quality difference and use χ_*DS*_ = χ_*PS*_ = 0.5 and χ_*DP*_ = 0.03. In this setting, the phase diagram should have several ideal features, i.e., the tie lines must have an angle *b* = −1, and for the dilution line that goes through the critical point, the fraction of the volume of the dextran-rich phase should extrapolate to *F*_*D*_ = 0.5.

In Figure 8a the SF-SCF predictions for the binodals of the phase diagrams are shown for the three distributions mentioned above. In this case, we did not obtain the spinodals and were unable to find an accurate value for the critical point. To compensate for this, we have attempted to approach the critical point a bit more closely than above (by considering larger system sizes so that computations could continue for systems with wider interfaces). In line with expectations, the critical composition of the systems shifts to higher polymer concentrations the lower is the average molecular weight. All our distributions have in common that there are chains of dextran that are longer than the length of the PEG chains (*N*_*P*_ = 550) and (importantly) significantly smaller than the PEG chains. The latter property correlates to the observation that the binodal branch on the high φ_*P*_ side is displaced away from the axis.^34^ The interpretation is that low molecular weight dextran components dissolve in the PEG-rich phase, even when the solvent volume fraction is significantly below the critical value. This effect is highest for the distribution with most short chains (linear distribution), and is weakest for the flat distribution because these have the fewest short chains. The nontrivial distribution of the bimodal case causes the binodal to be closer to the flat distribution case in strong segregation and more in between the flat- and linear distribution at week segregation.

**Figure 8:**
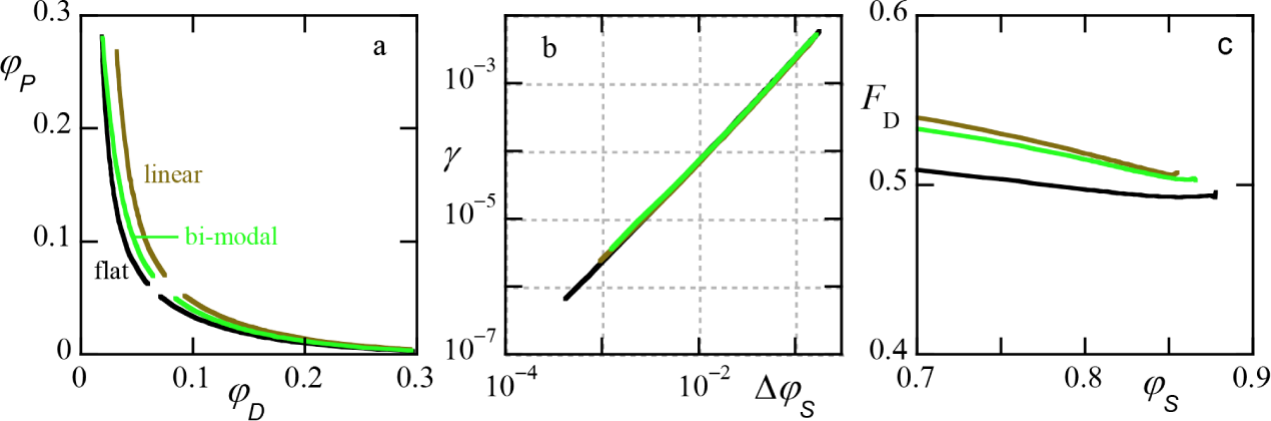
(a) Phase diagrams for ATPS, the effect of polydispersity. Only the binodals are presented. The dextran component has distributions’flat’,’linear’ and’bimodal’ (see text for info on the distributions) as indicated. (b) The corresponding interfacial tension as a function of Δφ_*S*_ in double logarithmic units. (c) The fraction of the volume taken by the dextran rich phase for the ideal dilution line going through the critical point. All three curves go within error to the value *F*_*D*_ = 0.5 when the volume fraction of solvent reaches the critical value. In the SF-SCF computation box the volume ratio dextran-rich phase to PEG rich phase is 1:1 (with less than 2% error). Parameters: *N*_*P*_ = 550. χ_*DP*_ = 0.03, χ_*DS*_ = χ_*PS*_ = 0.5.

From Flory–Huggins theory we know that when χ_*PS*_ = χ_*DS*_, the critical composition ratio $\frac{\varphi_{P}^{cr}}{\varphi_{D}^{cr}}=\sqrt{\frac{N_{D}}{N_{P}}}$ . Here, the dextran chains are polydisperse and it is of interest to know how this critical composition depends on the chain-length distribution. Inspection of Figure 8 indicates that the critical point moves to higher polymer concentrations, the lower the (number) average chain length. A more detailed analysis confirmed this. Defining $N_{D}^{\mathrm{eff}}$ as $\frac{\varphi_{P}^{cr}}{\varphi_{D}^{cr}}=\sqrt{\frac{N_{D}^{\mathrm{eff}}}{N_{P}}}$ , it was found that the effective chain length is proportional to the number-averaged and that for the three distributions tested, the proportionality constant is the same: $N_{D}^{\mathrm{eff}}=1.25N_{n}$ . This result is anticipated by the moment approach,^29–31^ and it suggests an important role of the osmotic pressure (which is related to the number-averaged chain length) in triggering phase segregation.

In line with recent findings^34^ also for the polydisperse systems the interfacial tension (cf. Figure 8b) still gives the expected power-law dependence. We remind the reader once again that we used the average water volume fraction over the two phases on the *y*-axis. The critical volume fractions of solvent $\varphi_{S}^{cr}\approx 0.87825,0.8672,0.8595$ for the flat, bimodal and linear distributions, respectively.

In Figure 8c the fraction of the volume taken by the dextran-rich phase *F*(φ_*S*_) is presented as a function of the volume fraction of solvent along a dilution line. All three curves in this figure are for ideal dilution lines that go through the respective critical composition (hence go to *F*_*D*_ = 0.5). Dilution lines that do not go through the critical composition invariably bend off to either zero or unity (not shown).

In SF-SCF calculations it is possible to control the position of the interface in the system by specifying the amount of the dextran component. Once the interface is placed, the volumes of the dextran-rich phase and PEG-rich phase are specified. We have iteratively adjusted the amount of the dextran component such that the ratio of the volumes of the dextran to the PEG-rich phase is set to fixed values. Of course, in all cases the interface remains further from the system boundaries than the width of the interface. This leaves a relatively small range of dextran/PEG volumes to be sampled. This type of calculations should not be confused with the volume ratio computed for systems along a specified dilution line. Such a result is extracted from the lever-rule once the phase diagram is available. Placing the interface explicitly in the SF-SCF computation, one can account for differences in partitioning of various chain lengths of the dextran component over the two phases. As the amount of each chain length in the system is specified by the parent distribution, the volume-ratio dependent partitioning will influence the molecular composition of both phases, and thus will influence the resulting phase diagram. Again, as targeted in the moment analysis,^29–31^ the cloud- and shadow curve are estimated when the volume of the minority phase is small compared to that of the minority phase.

Figure 9a shows that the phase diagram for the flat (weight) distribution case with specified volume-ratio of the two phases depends on this parameter. Inspection of this graph shows that the larger is the dextran-rich phase compared to that of the PEG-rich phase, the closer the binodal runs to the *y*-axis. We can understand this because the displacement of the binodal line with respect to the *y*-axis is a measure for how much dextran chains can dissolve in the PEG-rich phase. This amount is dominated by the short chains. If the PEG phase is small compared to that of dextran, the loss of short chains of the dextran phase will hardly affect its concentrations and then the cloud curve,^29,30^ is found. Hence, the concentration of small chains in the PEG phase is relatively high (the driving force to go to the PEG phase remains high) and this characterizes the shadow-curve.^29,30^ Alternatively, when the dextran phase is relatively small, the loss of the short chains that migrated to the large PEG-rich phase will reduce the concentration of the short ones in the dextran phase and this hampers the transport of these chains to the PEG phase. Thus, the concentration of dextran in the PEG phase is somewhat reduced: the binodal is closer to the *y*-axis.

**Figure 9:**
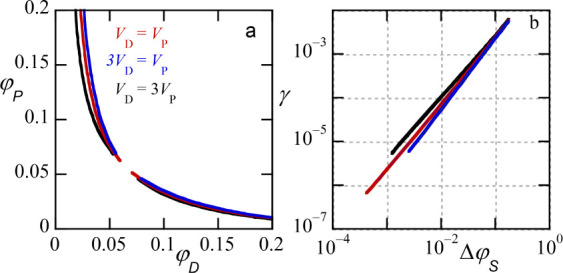
(a) Phase Diagram binodal for the flat (weight) distribution dextran component and monodisperse PEG with *N*_*P*_ = 550. The PEG volume fraction is on the *y*-axis and dextran volume fraction on the *x*-axis. (b) The interfacial tension as a function of Δφ_*S*_ in double logarithmic scale. Parameters: χ_*PD*_ = 0.03, χ_*PS*_ = χ_*DS*_ = 0.5. The SF-SCF computations are performed with the constraint that the volume-ratio of the PEG-rich phase and that of the dextran-rich phase is fixed as indicated.

If we focus on the critical region of these phase diagrams, it appears that the critical composition also slightly depends on the specified volume ratio. This is somewhat counterintuitive because at the true critical point the two phases become identical and their sizes cease to exist. The solution to this dilemma rests in the fact that the true critical point is only found for systems that have a specified volume ratio (in this case it must be 1:1, cf Figure 8b). So, only the red binodal, which has equal volumes of the two phases will have the true critical point as its limit where the two branches of the binodal meet. For the other two binodals, a’wrong’ phase-volume ratio is enforced, which is not compatible with the phase volumes at the critical point and therefore the two branches of the binodal cannot meet at the true critical point. We can only extrapolate to some’apparent’ critical point, which noticeably differs from the true critical point.

In Figure 9b we have plotted the interfacial tension versus Δφ_*S*_ in double logarithmic units. Interestingly, we find straight lines for all three cases, where it is understood that the critical $\varphi_{S}^{cr}$ implemented to find these straight lines slightly depends on the volume ratio. More specifically we have used the’apparent’ critical point data extracted from the binodals as discussed above. The slope of these linearized curves also deviate slightly from the 3/2 value. Only the curve with 1:1 volume ratio has the exact expected 3/2 slope.(

Intuitively, it is clear that in the case of polydispersity, the phase diagram depends on the volume of the phases that are used to compute the binodals. Smaller chains have a higher tendency to distribute over the system and thus go to the PEG-rich phase than the longer ones, and the more volume there is of the PEG-rich phase, the more small chains can’escape’ from the dextran-rich phase. Hence, the compositional drift must depend on the relative volumes of the two phases. It is possible to quantify this effect. To this end, we computed the number- and weight-averaged chain lengths of the dextran component in both the dextran- and PEG-rich phases. This also allowed us to evaluate the polydispersity index *I* = *N*_*w*_/*N*_*n*_ in both phases. In Figure 10 these quantities are shown for the’flat’ distribution as a function of the volume fraction of solvent in the system (average over the two phases).

**Figure 10:**
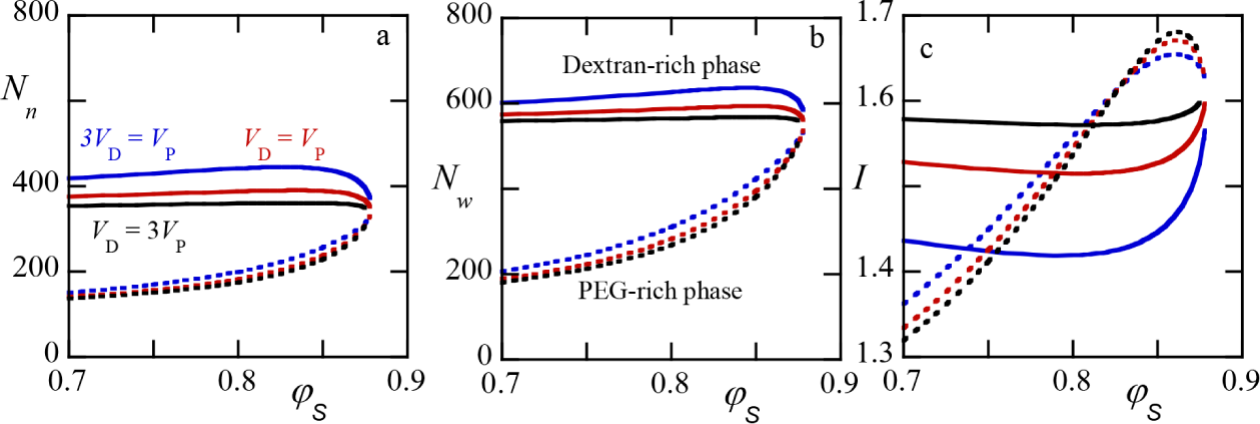
Quantification of the partitioning of dextran chains between the two phases for the flat (weight) distribution case. (a) The number-average chain length *N*_*n*_ in the dextran rich phase (solid lines), PEG-rich phase (dotted lines) as a function of the volume fraction of solvent φ_*S*_ (averaged over the two phases). The ratio between the volumes of the dextran and PEG rich phases are indicated. (b) The corresponding weight-averaged chain length *N_w_.* (c) The corresponding polydispersity index *I* = *N*_*w*_/*N*_*n*_. Parameters: *N*_*P*_ = 550, χ_*PD*_ = 0.03, χ_*PS*_ = χ_*DS*_ = 0.5.

The number-averaged chain length of dextran in the dextran-rich phase is much larger than that in the PEG-rich phase (cf. Figure 10a). This effect was already discussed above. Upon an increase of φ_*S*_ toward the critical point, we find that the number averages in both phases come together and at the critical point the average is simply that of the’parent’ distribution. For given φ_*S*_, the larger is the PEG-rich phase, the larger is the difference in averaged molecular weights of the dextran component in both phases. This is also true for the weight-averaged chain length (panel b). The weight-average invariably is larger than the number-average and thus the polydispersity index *I* (cf. panel c) is a value larger than unity. The lower this index, the more monodisperse in dextran the phase is. The lowest index is found far from the critical solvent concentration, that is, for strong segregation, in the PEG-rich phase. The highest polydispersity index occurs also in the PEG-rich phase in the weak segregation regime (relatively high values of φ_*S*_, close to the critical point). Again, at the critical point the data go to the imposed polydispersity index in the distribution. The polydispersity of the dextran-rich phase has the tendency to be less than the imposed one. This is caused by the loss of short chains and the relative enrichment of the longer ones. This reduction of polydispersity of the dextran chains in the dextran-rich phase is most pronounced when the PEG phase is relatively large.

We bring this subsection to a close by reporting on the density profiles of individual dextran components at the interface. In Figure 11 we present a set of volume fraction profiles for the flat-distribution at a volume fraction of the solvent in the PEG-rich phase of 0.7. The *z* = 0-value is at the Gibbs plane defined by the PEG component. Hence, the excess of PEG at the interface is set to zero. All dextran profiles are going from a relatively high value at negative coordinates to a much lower value at positive coordinates. Usually we expect tanh-like profiles.^22^ However, for smaller molecular weights (right-hand side of the figure), the profile goes through a small maximum, indicating some adsorption of this component at the interface. The reason for these nonmonotonous profiles may rest in the fact that long chains have a’depletion’-like distribution that scales with the square root of these chains (at least in these mean-field calculations). The smaller chains can fill this depletion zone such that the overall volume fraction profile of dextran remains close to constant until very close to *z* = 0. In line with this, the drop in the density profile (indicating that the density goes to the value in the PEG-rich phase) occurs for higher *z*-values for shorter chains. The solvent (water) also has a local maximum, which is significantly higher Δφ_*S*_ ≈ 0.01 than for any of the polymer components. We do not show this here because this is similar to and discussed in Figure 1b.

**Figure 11:**
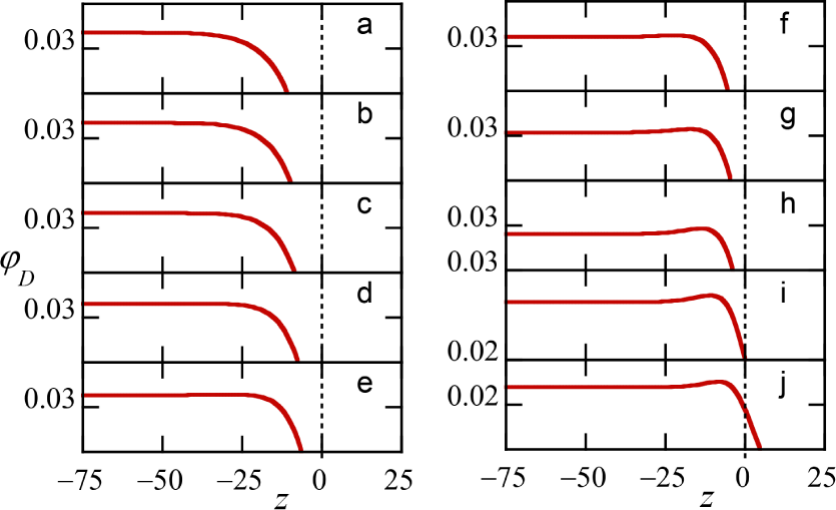
Set of zoomed-in volume fraction profiles of individual dextran components in the interfacial zone for the flat distribution. Parameters similar to Figure 10, specifically for $\varphi_{S}^{\beta }$ = 0.7 (relatively far from the critical point). All subplots have the same Δφ_*D*_ range of 0.01 on the *y*-axis. The vertical dotted line is set at the Gibbs plane (defined by the PEG component) set at *z* = 0. The interesting features occur in this case at negative values of *z*. (a) component *N*_*D*_ = 1000, (b) 900, (c) 800, (d) 700, (e) 600, (f) 500, (g) 400, (h) 300, (i) 200, and (j) 100.

The nontrivial distributions for the various components in the interface have been shown for φ_*S*_ = 0.7, that is, far from the critical point where the interface is relatively narrow. Closer to the critical point, that is for higher values of φ_*S*_ values, the nonmonotonic distributions no longer occur. This is expected because when the interface is wider than the size of the molecules, all molecules in the distribution must behave similarly.

### Relating SF-SCF Predictions to Experimental ATPS Phase Diagrams

Fitting of experimental phase diagrams is a challenge. One trivial issue is that experimental data are convoluted by experimental noise and uncertainties. The trouble with SF-SCF predictions is that these are of a mean-field type and fluctuations that broaden the binodal in the critical zone are not accounted for. Finally, polydispersity effects should be included. Hence, we cannot expect to find perfect matches between theory and experiments. Therefore, our goal is to find order-of-magnitude values for SF-SCF parameters that can fit the experiments reasonably closely.

Interestingly, Diamond and Hsu^5^ have tabulated binodal data for a number of dextran-water-PEG systems, which (in our opinion) are of very high accuracy and internal consistency. This data set is used to find estimates for the SF-SCF parameters. Several pragmatic choices had to be made to implement the comparison. (i) The first issue is that the phase diagram data are presented in %w/w, while in SF-SCF one needs volume fractions. We know that the densities of dextran and PEG are slightly above 1 g/mL, but we decided to simply divide by 100 to translate the %w/w to volume fractions. The systematic error caused by this is probably within the accuracy of the whole procedure. (ii) We need to translate the molecular weights of dextran- and PEG samples used to the degree of polymerization. The dextran is polydisperse and we know that the number-averaged molecular weight is important for the phase diagram. Diamond and Hsu^5^ used dextran fractions with number-averaged molecular weights of 234,200, 38,400 and 24 400 g/mol. These were reduced to order of magnitude-like numbers *N*_*D*_ = 500, 150, and 100 using two to four sugar units to form a statistical segment. The authors used PEG molecular weights 20,000, 8,000, and 3,400 which we translated into *N*_*P*_ = 100, 40, and 20 units, respectively, again using about four real segments to be a statistical segment (iii) The authors used so-called tie line experiments and determined the composition of the coexisting phases (by polarimeter for the dextran, and with refractive index measurements for overall polymer content- using pycnometry to convert from w/v into %w/w). From the data the slopes of the tie lines were obtained. Inspection of their results indicated that for lower molecular weight PEG systems the slopes were slightly less negative than for the higher PEG molecular weights. This indicated that the difference χ_*DS*_ – χ_*PS*_ is larger for long PEG chains than for short ones. This is in line with data from the literature for the solvent quality of PEG. The χ_*PS*_ tends to be higher when the molecular weight is higher and vice versa. We implemented this as follows. We fixed χ_*DS*_ = 0.48 throughout the computations and set χ_*PS*_ = 0.44 for *N*_*P*_ = 100, χ_*PS*_ = 0.3 for *N*_*P*_ = 40 and χ_*PS*_ = 0.25 for *N*_*P*_ = 20. (iv) We choose to fix χ_*DP*_ = 0.2, that is, the strength of the driving force for segregation, to a fixed value irrespective of the molecular weights used in the system.

Nine phase diagrams were fitted and their results are presented in Figure 12. The data points are obtained from experiments and the continuous line are the SF-SCF predictions. The comparison is satisfactory. Although the strength of the minor driving force compared to the major driving force changes significantly between the systems, we were able to locate the position of the critical point with reasonable accuracy. The trends of the slopes of the tie lines were also matched. As our interest is more in the high molecular weight systems (panels g, h and i), we have paid a bit more attention to these cases.

**Figure 12:**
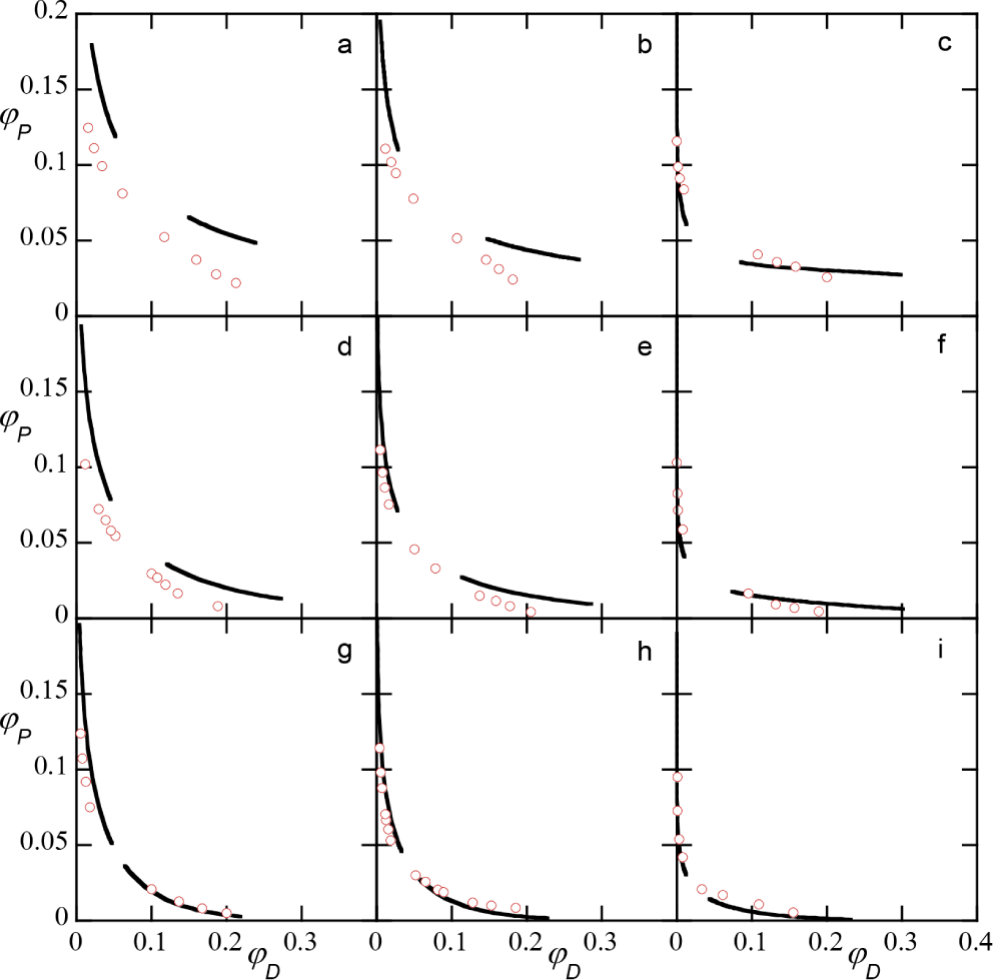
SF-SCF fits (solid lines) of experimental data (red dots). Experimental systems:^5^ dextran (number-averaged) Mw: 234 200 g/mol (c, f, i), 38,400 (b, e, h), 24 400 g/mol (a, d, g). PEG Mw: 20 000 g/mol (g, h, i), 8000 g/mol (d, e, f), 3400 g/mol (a, b, c). Corresponding SF-SCF parameters: *N*_*D*_ = 500, 150, 100, *N*_*P*_ = 100, 40, 20. Interaction parameters: χ_*DP*_ = 0.2, χ_*DS*_ = 0.48. PEG-water interactions are taken to be molecular weight dependent: χ_*PS*_ = 0.44, 0.3, 0.25 for the *N*_*P*_ = 100, 40, and 20, respectively.

Some observed deviations were expected. For low PEG molecular weight systems the theoretical phase diagram is more curved than the experimental data indicates. We attribute this to the failure of the mean-field theory. Ignoring fluctuations causes the phase diagram to be too curved in the critical region. In SF-SCF, the phase diagrams a, b, c, invariably have a significant concentration of PEG in the dextran-rich phase (simply because the molecular weight is low), whereas the data point suggests a lower PEG solubility in the dextran-rich phase. This is only’corrected’ with a much higher value for χ_*PS*_, but then the critical point was found to be too low. Hence, we could not satisfy both issues at the same time. We opted to have the critical point in the proper range, and accepted the relative overprediction of the solubility of PEG in the dextran-rich phase. Again we believe that this problem can be traced to the mean-field character of the SF-SCF predictions.

For higher molecular weight systems the match of theoretical and experimental phase diagrams is perhaps even better than expected. This does not mean that we have perfectly hit the proper translation of experimental data to modeling parameters. Small variations of the parameters may have done a comparable job.

In our laboratory, we have determined a phase diagram for dextran 150,1000 g/mol, PEG 20 000 g/mol using cloud point titrations (addition of the minority polymer component to an one-phase system until a minority phase emerges as seen by a persistent increase of the turbidity) and dilution experiments (adding water to a two-phase system until the phase boundary disappears).^36^ Compared to the set of phase diagrams presented in Figure 12, our system is between the systems presented in panels (h) and (i). We have chosen *N*_*D*_ = 300 to model our own phase diagram; hence we have ignored the polydispersity as yet. As this system will be used extensively for the SF-SCF modeling of wetting and capillary suspension experiments in future work, we will analyze this system in slightly more detail.

The fitting of our phase diagram (cf. Figure 13a) is satisfactory. Data points follow the predicted solid line (binodal) to a good extend, albeit that there are a few data points in the lower left corner that definitely fall outside the predictions. We believe that this can be attributed to polydispersity effects. More specifically, when there is a minority high molecular weight component in the system, we can anticipate such effect. In this case, the closer one gets to the critical point, the higher is the molecular weight-average of the dextran rich phase and compared to the ternary case the tie lines will be even less tilted (*b*-values are less negative).

**Figure 13:**
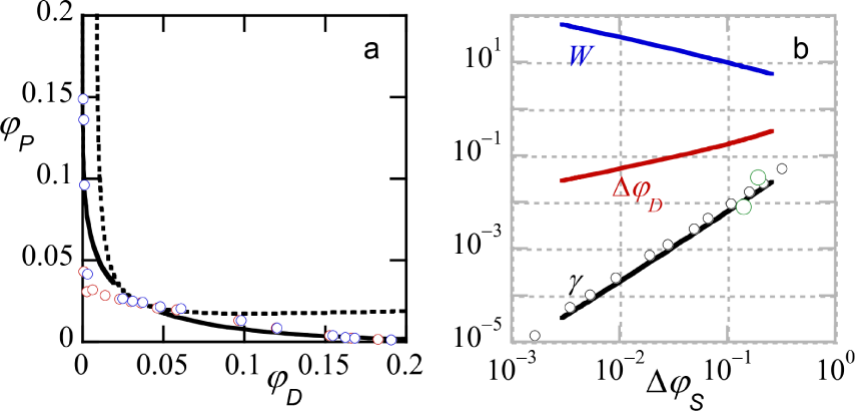
(a) Phase diagram for dextran Mw 150 000 g/mol, PEG Mw 20 000 g/mol. SF-SCF fits (binodal solid lines, spinodal dotted line), experimental data (red and blue points are measured by two independent individuals). Modeling parameters as in Figure 12, *N*_*D*_ = 300. (b) The width *W*, the density difference Δφ_*A*_, the interfacial tension γ (in units of *k*_*B*_ *T*/*b* ^2^) as a function of Δφ_*S*_, with critical value $\varphi_{S}^{cr}\approx 0.942$ in double logarithmic units. On the interfacial tension curve the points represent measurements with the spinning drop method and presented in dimensionless units. The bigger (green) dots are for the same system as presented in panel (a). The black dots are for a slightly different system from the literature,^35^ i.e., dextran Mw between 300 and 400 kg/mol and PEG Mw 7 kg/mol.

The dotted line is the predicted spinodal. In the model, the critical point is located where the binodal and spinodal touch. Experimentally, it remains uncertain where the critical point is exactly located. The modeling helps to pinpoint its location somehow, although the mentioned polydispersity effect still contributes to the uncertainty of its location.

For future comparison, we have plotted the interfacial width, the density difference, and the interfacial tension as a function of $\Delta \varphi_{S}=\varphi_{S}^{cr}-\left(\varphi_{S}^{\beta }+\varphi_{S}^{\alpha }\right)/2$ in double logarithmic units. As expected, the curves are straight lines, indicating that $\varphi_{S}^{cr}=0.942$ in this case. Few of our own experimental interfacial tension data points are presented as well. These interfacial tensions were obtained by the spinning drop method and converted to dimensionless units by the conversion factor mentioned above. One should understand that the measurements are far from trivial, and the number of points is too low to say with confidence that there is a good correspondence. However, clearly SF-SCF predictions give the proper order of magnitude for the interfacial tension. More accurate interfacial tension data are available in the literature,^6,35^ and these were turned to dimensionless units and inserted in Figure 13b (black dots) even though these data were for slightly different MWs for the dextran and PEG polymers.^6^ The correspondence between experiments and theory indicates that for these ATPS the mean-field scaling exponent of 3/2 is followed to a good extent. Only very close to the critical point we expect deviations from the mean-field scaling due to the neglect of fluctuations (this was suggested in ref 6) and polydispersity effects that show up most in the critical region.

Ideally, theoretical modeling should assist experimentalists in underpinning experimental finding or explaining trends in data. In so-called tie line experiments one can find the concentrations of coexisting phases and from this, find the slope of the tie line. Another interesting observable is the fraction of the volume taken by the dextran-rich phase *F*_*D*_ along a dilution line. This quantity is informative about the location of the critical point. It was discussed above that there is only one dilution line for which *F*_*D*_ → 0.5, namely the dilution line that goes exactly through the critical point. These two observables are collected in Figure 14.

**Figure 14:**
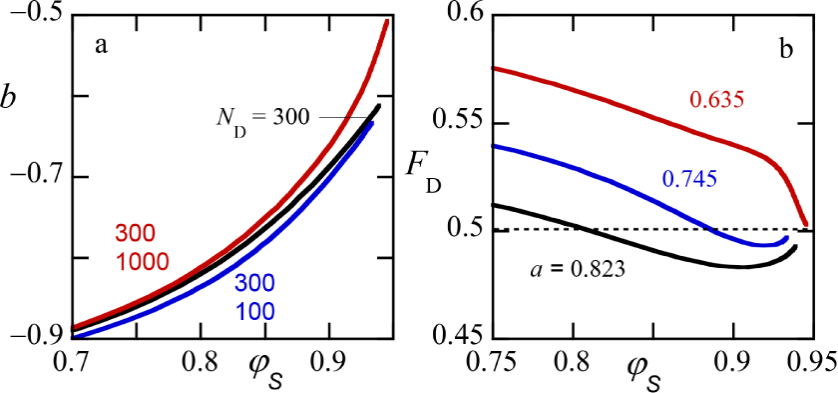
(a) SF-SCF predictions for the slope of the tie line *b* as a function of volume fraction of solvent φ_*S*_ in the system: black line correspond to the theoretical binodal of Figure 13, i.e., for the ternary system; red line is for dextran consisting of two components with *N*_*D*_ = 300 and 1000 with ratio 3:1; blue line is for dextran consisting of two components *N*_*D*_ = 300 and 100 with ratio 3:1. (b)­The fraction of the volume in the system taken by the dextran rich phase *F*_*D*_ plotted as a function of the volume fraction of solvent $\varphi_{S}=\left(\varphi_{S}^{\alpha }+\varphi_{S}^{\beta }\right)/2$ in the system for the ideal dilution lines that go through the critical points (all curves extrapolate to 0.5). Lines have same color code as in panel (a) and the slope of the ideal dilution line *a* is indicated. In the systems that are bidisperse (red and blue lines) the volume ratio of the two phases is 1:1.

In panel a) of this figure with the black line the prediction is given for the dependence of the slope of the tie line *b* that corresponds to the SF-SCF prediction of the phase diagram presented in Figure 13a, that is, for the ternary system. In line with the trend for *b* reported in Figure 7a the slope deviates from −1 (it is significantly less negative) because there is a solvent-quality difference for the two polymers. Again, we see that the tie lines are not parallel and become less steep when the critical point is approached. When the dextran component is bidisperse, that is, when there is besides the major *N*_*D*_ = 300 component a minor component with either a longer (red-line: *N*_*D*_ = 1000) or shorter (blue line: *N*_*D*_ = 100) chain is added in a ratio 3:1, the value of *b* is noticeably different. In the first case, the difference is particularly large near the critical point; in the second case the most important difference is relatively far from the critical point. Again, these trends are easily explained from the partitioning effect. The longest polymers are the last ones that remain separated; the shortest chains leak out to the PEG phase first.

The prediction of the phase volume *F*_*D*_ along the ideal dilution line that goes through the critical point, is shown in Figure 14b. The pure ternary system (black line) has a larger dextran-rich phase far from critical, while near critical the PEG-rich phase is largest before the two phases become of the same size (at the critical point). There is a surprisingly large effect of admixing a longer or shorter chain to this system, and this explains why it is difficult to capture the experimental results for this quantity. When a longer chain (red line) is added (in 1:3 ratio), the dextran-rich phase is the largest at all solvent concentrations. Interestingly, when a smaller chain is added instead, (blue line) the *F*_*D*_(φ_*S*_) dependence is in between these cases.

When we consider the slope *a* of the ideal dilution lines, we see that the bidisperse cases both have a value of *a* that is lower than the pure ternary system (black line). This means that the ratio PEG-to-dextran at the critical point (φ_*P*_/φ_*D*_)^cr^ tends to be lower when the dextran chains are polydisperse. It is therefore expected that the SF-SCF prediction of the phase diagram based on a pure ternary system underestimates the PEG/dextran ratio at the critical point. Returning to the phase diagram of Figure 13a, the data points in the lower left corner not only indicate that there are longer dextran chains in the system (most likely as minority components), but also that the critical point of the real system must be at lower PEG/dextran ratios than predicted by the SF-SCF result (which was based on the pure ternary system).

## Discussion and Conclusions

Aqueous two-phase systems are oil-free alternatives for formulations of, e.g., emulsions in foods.^3,12^ They are of interest to study fundamental issues in segregative phase behavior of polymeric systems, as the water content is a very convenient control parameter.^6,8,35^ There are of course intricacies. For example, the main system that is studied contains dextran and PEG as the main polymeric components, and these are slightly thermosensitive (so temperature matters as well), the polymers are polydisperse: the approximation of the system as a ternary system is relatively good far from the critical point, but not very good near the critical zone where partitioning becomes very important. Finally, dextran is nonideal because it is known to be branched. Branching is not addressed in the current paper because on the Flory–Huggins level there is no effect of branching. On the SF-SCF level, there are small effects from branching as it influences the width of the interface and the interfacial tension, but these effects are secondary. As we have shown, theoretical modeling is possible for this system and experimental work in this field will hopefully benefit from it.

SF-SCF theory is useful to model aqueous two-phase systems. Even though the phase diagram can already be found from the Flory–Huggins model, we believe that additional information about the system (the width of the interface, the interfacial tension) and the partitioning of chains in the polydisperse systems give useful additional information which can inspire experimentalists.

We have identified modeling parameters for which segregative interactions lead to a closed binodal and to the open binodals. The first happens when the major driving force is weak and a significant difference in solvent quality exists. The open binodals must be considered as the most experimentally accessible ones, and occur when the major driving force, i.e., the repulsion between the two chains, is significant. We found that when there is no difference in solvent quality, the tie lines all have a slope of *b* = −1, irrespective of whether or not the critical point is on a dilution line *a* = 1 (this occurs only when the chain lengths are equal). When water prefers PEG over dextran, that is, when there is a difference in solvent quality, we have shown that the tie lines no longer are perfectly parallel. The dilution line that goes through the critical point has the nice feature that at the critical point the phase volumes are equal.

Polydispersity effects complicate the evaluation of the binodal. The fundamental reason for this is that the binodal becomes a surface in a multidimensional space. Projecting this on a two-dimensional plot with overall volume fraction of PEG versus volume fraction of dextran, presents the problem that the resulting binodal becomes a function of the volume ratio PEG/dextran that is used in determining the binodal values (e.g., through tie-line experiments). The two coexisting phases carry different fractions of the polydisperse chains with different concentrations. One can find either more polydisperse subphases or more monodisperse subphases, depending on whether or not the system is near or far from the critical region.

Fitting of experimental phase diagrams is a challenge. Invariably near the critical region the SF-SCF predictions cannot exactly mimic the experiments because of the mean-field approximation. This is more of an issue for the low molecular weight systems, because then fluctuations are important relatively far from the critical point. For longer polymers, the critical zone does not extend far from the critical point, and the SF-SCF model is doing a better job. However, polydispersity effects can still lead to strong differences between theoretical and experimental binodals.

The modeling parameters that approximately reproduced our experimental phase diagram will be used in future publications to model wetting and capillary condensation phenomena for these systems.

## Supplementary Material



## Notes

Basics of self-consistent field theory with discretization scheme of Scheutjens and Fleer with a focus on the machinery and its premises (PDF)
