# Supplementary material for: Self-consistent Field Analysis of Segregative Aqueous Dextran–Polyethylene Glycol Solutions: (1) Bulk Phase Diagrams
Source: J Phys Chem B. 2025 Jun 19;129(26):6632–45. doi: 10.1021/acs.jpcb.5c01284 (PMC12235633; doi:10.1021/acs.jpcb.5c01284)
Supplement: Supplementary file 1 [file jp5c01284_si_001.pdf]

Self-Consistent Field Analysis of Segregative Aqueous Dextran - Polyethylene Glycol Solutions: (1) Bulk Phase Diagrams

F.A.M. Leermakers,*,† L. Ruiz-Martínez,† S.D. Stoyanov,‡ and J. van der Gucht†

†*Physical Chemistry and Soft Matter, Wageningen University, Stippeneng 4, 6708 WE Wageningen, the Netherlands*

‡*Food, Chemical, and Biotechnology cluster, Singapore Institute of Technology, 10 DoverDrive, Singapore, 138683 Singapore*

E-mail: frans.leermakers@wur.nl

Abstract

The goal of this extra information is to sketch the main features of the SF-SCF modeling. For even more details, we refer to the literature.[1–6]

The SF-SCF modeling starts with a mean-field free energy functional $F([\varphi],[u],\alpha)$ that features for each segment type X complementary distributions of the volume fraction $\varphi_X(z)$ and the segment potential $u_X(z)$.[7] The square-brackets imply the presence of the quantity for all segment types and all coordinates. In this paper, we use a planar coordinate system with z as spatial coordinate, in which gradients (of the volume fractions and potentials) are expressed. A mean field approximation is applied in planes perpendicular to the z-coordinate, that is, in the x-y plane. The extremization of this free-energy functional leads to self-consistent field relations that can be solved numerically. To facilitate this, a discretization scheme is used. In such a scheme, the space is discretized and visualized as a system of

lattice sites (with fundamental length b as the size of each element). Then the z-coordinate is redefined as a layer number, that is, $z \equiv \frac{z}{b} = 1,\ 2,\ \cdots,\ M$, where M specifies the total number of lattice layers used in the computations. Polymer chains (type index i) are assumed to be composed of segments $s = 1,\ 2,\ \cdots,\ N_i$, where N_i is the degree of polymerization of chain number i. Scheutjens and Fleer borrowed the idea from Flory-Huggins theory that a segment fits in a lattice site. So, also the segments are assumed to be of length b. Typically, the freely-jointed chain approximation is implemented in the computations. In this approach, two consecutive segments along the chain occupy neighboring lattice sites, and two consecutive bonds in the chain can have random mutual orientations. This means that bond-bond correlations are ignored, implying that segments that are not neighbors may end up in the same lattice site. This excluded-volume problem, is counteracted by an incompressibility constraint which is implemented in a layer fashion: in each layer the volume fractions summed over all molecules must equal unity: $\sum_i \varphi_i(z) = 1$ for all z-values. Typically, we express all extensive quantities per unit area. We use a Lagrange field $\alpha(z)$ to control this incompressibility property of the system. Hence, above the free energy functional is having these α-values as its parameters as well.

Again, the optimal free energy is found numerically by an iterative search. When the so-called SCF-solution is known we can compute the grand potential $\Omega = F - \sum_i \mu_i n_i$, where n_i is the number of molecules of type i in the system, and μ_i is the chemical potential of molecule i. This chemical potential is a function of the bulk concentrations only and we use the Flory-Huggins equations for this. The grand potential,[4] which here may be interpreted as the interfacial tension, can be written as $\Omega = \sum_z \omega(z)$ where $\omega(z)$ is the grand potential density

$$\omega(z) = -\sum_i \frac{\varphi_i(z) - \varphi_i^b}{N_i} - \alpha(z) - \frac{1}{2}\sum_X \sum_Y \chi_{XY} \left(\varphi_X(z)\langle\varphi_Y(z)\rangle - \varphi_X^b \varphi_Y^b\right) \qquad (1)$$

Again the SCF machinery is used to find the quantities that are needed to compute the grand

potential densities. In short, the segment potentials are a function of the volume fractions. So when it is assumed that the volume fractions are known, the potentials are valuated by

$$u_X(z) = \alpha(z) + \sum_Y \chi_{XY}\left(\langle\varphi_X(z)\rangle - \varphi_X^b\right) \tag{2}$$

Here χ is the Flory-Huggins interaction parameter, defined as

$$\chi_{XY} = \frac{Z}{2k_BT}\left(2U_{XY} - U_{XX} - U_{YY}\right) \tag{3}$$

where k_BT is the thermal energy, Z is the lattice coordination number (e.g., $Z = 6$ for a simple cubic lattice and $Z = 4$ for a hexagonal lattice). U_{XY} is the nearest-neighbor contact energy contribution when two neighboring sites are occupied by an X and an Y-type segment. From its definition it is easily seen that the χ parameter is only non-zero when X and Y are different segment types. As the χ contains the thermal energy it is easily seen that the segment potentials u_X (cf Eqn 2) are in fact dimensionless (i.e. they are expressed in units of k_BT). Again, the α-values are coupled to the incompressibility constraint:

$$\sum_i \varphi_i(z) = \sum_X \varphi_X(z) = 1 \tag{4}$$

summing the volume fractions over the molecules or over the segment types must be the same and add-up to unity. If this (during the iterations) is not the case at some coordinate z', the value of $\alpha(z')$ is modified by $\alpha(z') \equiv \alpha(z')^{\text{new}} = \alpha(z')^{\text{old}} + \eta(\sum_i \varphi_i(z) - 1)$, where η is a regularization parameter between 0 and 1 used to prevent oscillations of α (typically a value of $\eta = 0.5$; the larger the chains, the smaller should be this value). In equation 2 the angular brackets are needed to properly account for the number of contacts when the densities are spatially inhomogeneous. It signals a three-layer average; for any z-dependent quantity $\psi(z)$

$$\langle\psi(z)\rangle \equiv \lambda_{-1}\psi(z-1) + \lambda_0(z)\psi(z) + \lambda_1\psi(z+1) \tag{5}$$

In a planar cubic lattice $\Lambda_a - 1 = \lambda_1 = 1/6$ and $\lambda_0 = 4/6$; in a hexagonal lattice the values are 1/4 and 1/2 respectively. Finally, in Eqn 2 the volume fraction of X in the bulk is given by φ_X^b. Below, where we go into some details on how computations are done, we will define what we mean with the bulk more precisely.

The other half of the self-consistent field method specifies how one can compute the volume fractions when the values of the potentials are known. For the freely-jointed chain model, the procedure that is followed is known as the propagator formalism. The first step is to convert the potentials to free segment weighting factors

$$G_X(z) = \exp\left(-u_X(z)\right) \tag{6}$$

$$G_i(z,s) = \sum_X G_X(z)\delta_{i,s}^X \tag{7}$$

where we recognize that the weighting factors are of a Boltzmann weight type. The second variant implies that, when segment s of molecule i is of type X (then $\delta_{i,s}^X = 1$, else it is zero), the statistical weight is given by the $G_X(z)$. We recall that the potentials are already normalized by k_BT and thus we do not need this term in the exponent of the Boltzmann weight. Next, so-called end-point distribution functions $G_i(z,s|z',s')$ are defined. This quantity will contain the statistical weight of all possible and allowed 'walks' (conformations) that start with segment number s' at layer z' and end with segment s at coordinate z. In the propagator formalism an integrated variant of the end-point distributions occurs, which is defined as:

$$G_i(z,s|s') \equiv \sum_{z'} G_i(z,s|z',s') \tag{8}$$

More specifically, we use two variants: in the forward propagator $s' = 1$ and in the backward one $s' = N_i$. The two propagators are initiated by understanding that a walk with length 1 must have the weight given by the free segment distribution function. Hence

$$G_i(z,1|1) = G_i(z,1) \tag{9}$$

$$G_i(z, N_i|N_i) = G_i(z, N_i) \tag{10}$$

and all other end-point distribution functions follow recursively by

$$G_i(z, s|1) = G_i(z, s)\langle G_i(z, s-1|1)\rangle \tag{11}$$

$$G_i(z, s|N_i) = G_i(z, s)\langle G_i(z, s+1|N_i)\rangle \tag{12}$$

Here the angular brackets again define a three-layer average as above in Eqn 5. Inspection of Eqns. 11,12 easily reveals why these equations are also known as propagators. The top one is the 'forward' propagator: end-point distribution functions are computed going from $s-1$ to s and the second one is the 'backward' propagator as it goes from $s+1$ to s to compute the relevant statistical weights. The propagator equations 11,12 can be shown to be the discrete version of the Edwards diffusion equation often used to describe polymer properties.[8]

The volume fractions are now computed using the so-called composition law. The procedure calls for a combination of two complementary end-point distribution functions:

$$\varphi_i(z, s) = C_i \frac{G_i(z, s|1)G_i(z, s|N_i)}{G_i(z, s)} \tag{13}$$

The division by $G_i(z, s)$ corrects for the fact that the statistical weight for segment s is needed only once: indeed, as it is included in both $G_i(z, s|1)$ and $G_i(z, s|N_i)$, we need to correct it. We have two equations for the normalization of the volume fraction distributions C_i: (i) one we can use when the bulk concentration of a component is known (grand canonical calculations) and (ii) the other one when the number of molecules n_i is specified (canonical calculations):

$$C_i = \frac{\varphi_i^b}{N_i} \quad \text{grand canonical calculations} \tag{14}$$

$$C_i = \frac{n_i}{q_i} \quad \text{canonical calculations} \tag{15}$$

Here q_i is the chain partition function which follows from integrating the (final) end-point distribution functions:

$$q_i = \sum_z G_i(z,1|N_i) = \sum_z G_i(z,N_i|1) \tag{16}$$

Volume fractions $\varphi_i(z)$ follow from summation over all segment ranking numbers s, $\varphi_i(z) = \sum_s \varphi_i(z,s)$. The volume fraction per segment type results from $\varphi_X(z) = \sum_i \varphi_i(z,s)\delta^X_{i,s}$. The bulk volume fractions φ_i^b can be found from equating the two variants for C_i (cf. Eqn 14,15. In the calculations we always have one component k for which the normalization

$$C_k = 1 - \sum_{i \neq k} \varphi_i^b \tag{17}$$

This guarantees that the reference 'bulk' is at all times incompressible, as it should.

From the above, we have seen that the volume fractions are computed from the potentials and *vice versa* the potentials are computed from the volume fractions. In principle, we can feed the computed values for one quantity into the calculations of the other, until a stationary point is reached. Meanwhile, as mentioned before, α-values are updated until for the coordinates they refer to the incompressibility condition is met. To guarantee the convergence toward an SCF solution, we usually need to introduce regularization parameters for the segment potentials, similarly as for the α-value updates.

The mentioned iterative procedure will converge (slowly). In practice, however, we use a more complex newton-like iteration scheme that gives convergence of the equations in a reasonable number of iterations (10^2 to 10^3).[4] In all cases the iterations continue until there are at least 8 significant digits for both the potentials (including α-values) and the volume fractions. Once the SCF-solution is known, we can compute the grand potential (cf. Eqn 1) to high accuracy, as the volume fractions of all components as well as the values α are known.

The calculation of the two-phase state of aqueous two-phase systems proceeds as follows. We choose a planar geometry with $M = 1000$ layers of lattice sites and select the

hexagonal lattice type ($Z = 4$). At the system boundaries we apply reflecting boundary conditions (needed when the angular brackets (cf Eqn 5) are to be implemented near the system boundary)

$$\Psi(0) = \psi(z) \tag{18}$$

$$\psi(M+1) = \psi(M) \tag{19}$$

so that adverse boundary effects are absent (that is when the interface is far from the system boundaries).

Typically, by way of a suitable initial guess, the dextran-rich phase is placed at the lower values of the coordinate system (phase α) and the PEG-rich phase is at the higher values of the coordinate system (phase β). The latter phase coincides with what we will call the 'bulk'. The amount of dextran (that is n_D is typically specified as an input quantity (that is, the calculation type is 'canonical' for this component). This number is adjusted accordingly when the interface appears too close to the system boundary. The volume fraction of water is specified by φ_S^b (that is, the value of the volume fraction of water in the PEG-rich phase is fixed). So, for the water component, we have the grand canonical computation strategy. The remaining PEG component is normalized using the condition given by Eqn 17. Hence, the amount of PEG in the system will adjust until the system is exactly filled to capacity (both the solution and the reference 'bulk').

When in some calculations the volumes of phase α and β must be controlled, we perform consecutive calculations and adjust the amount of the dextran component(s) until the interface is at the desired location to a specified accuracy (mostly relevant for polydisperse systems).

References

(1) Scheutjens, J. M. H. M.; Fleer, G. J. Statistical theory of the adsorption of interacting chain molecules. I. Partition function, segment density distribution and adsorption isotherms. *J. Phys. Chem.* **1979**, *83*, 1619–1635.

(2) Scheutjens, J. M. H. M.; Fleer, G. J. Statistical theory of the adsorption of interacting chain molecules. II. Train, loop, and tail size distribution. *J. Phys. Chem.* **1980**, *84*, 178–190.

(3) Fleer, G. J.; Cohen Stuart, M. A.; Scheutjens, J. M. H. M.; Cosgrove, T.; Vincent, B. *Polymers at interfaces*; Chapman and Hall, London, 1993.

(4) Evers, O. A.; Scheutjens, J. M. H. M.; Fleer, G. J. Statistical thermodynamics of block copolymer adsorption. 1. Formulation of the model and results for the adsorbed layer structure. *Macromolecules* **1990**, *23*, 5221–5232.

(5) De Lange, N.; Kleijn, J. M.; Leermakers, F. A. M. Structural and mechanical parameters of lipid bilayer membranes using a lattice refined self-consistent field theory. *Phys. Chem. Chem. Phys.* **2021**, *23*, 5152–5175.

(6) Leermakers, F. A. M.; Léonforte, F.; Luengo, G. S. Structure and Colloidal Stability of Adsorption Layers of Macrocycle, Linear, Comb, Star, and Dendritic Macromolecules. *Macromolecules* **2020**, *53*, 7322–7334.

(7) Varadharajan, R.; Leermakers, F. A. M. The physics of microemulsions extracted from modeling balanced tensionless surfactant-loaded liquid–liquid interfaces. *J. Chem. Phys.* **2020**, *152*, 094902.

(8) Edwards, S. F. The statistical mechanism of polymers with excluded volume. *Proc. Phys. Soc.* **1965**, *85*, 613–624.
